# Assessment of isomiR Discrimination Using Commercial qPCR Methods

**DOI:** 10.3390/ncrna3020018

**Published:** 2017-03-24

**Authors:** Rogan Magee, Aristeidis G. Telonis, Tess Cherlin, Isidore Rigoutsos, Eric Londin

**Affiliations:** Computational Medicine Center, Thomas Jefferson University, 1020 Locust Street, Philadelphia, PA 19107, USA; Rogan.Magee@jefferson.edu (R.M.); Aristeidis.Telonis@jefferson.edu (A.G.T.); Tess.Cherlin@jefferson.edu (T.C.); isidore.rigoutsos@jefferson.edu (I.R.)

**Keywords:** isomiR, miRNA, qPCR, short non-coding RNA

## Abstract

We sought to determine whether commercial quantitative polymerase chain reaction (qPCR) methods are capable of distinguishing isomiRs: variants of mature microRNAs (miRNAs) with sequence endpoint differences. We used two commercially available miRNA qPCR methods to quantify miR-21-5p in both synthetic and real cell contexts. We find that although these miRNA qPCR methods possess high sensitivity for specific sequences, they also pick up background signals from closely related isomiRs, which influences the reliable quantification of individual isomiRs. We conclude that these methods do not possess the requisite specificity for reliable isomiR quantification.

## 1. Introduction

MicroRNAs (miRNAs) are a well characterized family of short non-coding RNAs [1,2]. These regulatory molecules are cleaved from premature transcripts by Dicer family proteins to form ~22 nucleotide (nt) molecules. They function to negatively regulate gene expression of messenger RNAs (mRNAs) in a sequence dependent manner [1,3], to inhibit translational initiation [4] via disruption of cap–tail interactions [5,6], and to promote exonuclease-mediated mRNA degradation [7,8]. It is now appreciated that nearly all genes and cellular programs important for the development and progression of complex diseases are regulated by miRNAs [9].

Traditionally, each miRNA precursor was believed to give rise to a single mature miRNA sequence, but recent work [10,11,12,13,14] has revealed a more complex system at play. The use of short RNA sequencing has revealed that a miRNA precursor constitutively produces a cloud of mature miRNAs from a given precursor arm, instead of only one. These isomiRs differ from the archetypal sequence (the one annotated in public reference databases such as miRBase [2]) on their 5′- and/or -3′ end. Our previous analysis of 452 deep sequencing datasets from the GEUVADIS project [15] showed that isomiR profiles are able to classify individual samples based on characteristics, such as: sex, race, and population [11]. More recently, we showed that isomiRs can distinguish amongst tissue type and disease subtypes and that isoforms of the same miRNA can target largely non-overlapping groups of mRNAs [10,16].

While the functional importance of isomiRs has not been fully understood, the shift on their 5′-end alters the miRNA seed sequence, resulting in changes in target profiles. Evidence for the functional consequences of this shift is accumulating. In [10], we showed that transfection of miR-183-5p isomiRs −2|−2 and +2|+2 into MDA-MB-231 cells shows a less than 15% overlap in targeted mRNAs, as compared to the archetypal miR-183-5p 0|0 isomiR. A more recent study [13] demonstrated that the different seed sequences of miR-140-3p 5′-isomiRs functionally target distinct downstream genes that the archetypal sequence lacks the ability to regulate. In the context of type 2 diabetes, isomiRs of the miR-375 locus target an overlapping, but distinct complement of beta cell genes, as compared to the archetypal sequence [17]. Finally, a third group also recently demonstrated that miR-142-3p 5′-isomiRs have a diverging, yet overlapping effect on actin dynamics in immortalized human microvascular endothelial cells [12]. These studies suggest that isomiRs are not only an actual feature of a miRNA locus, but that their presence can considerably increase the repertoire of genes regulated by a specific locus. By extension, isomiRs are expected to play important roles in disease etiology and, thus, their functions warrant detailed studies. To this end, accurate detection of isomiRs and accurate quantification of their abundance is vital.

Among the many current technologies [18], RNA sequencing is the only technique that affords such detection and quantification in an unbiased manner. However, owing to its high cost and the complexity of the required computational analysis, this approach is not widely accessible. Moreover, it is often necessary to characterize only one, or a small group of isomiRs, rather than the full complement of isomiRs within the sample. Multiple detection methods are available to efficiently and accurately quantify miRNAs [18], but these approaches have not been tested for their ability to quantify specific isomiRs. Therefore, there is currently an unmet need to determine how effective these approaches are in distinguishing varying isomiRs.

Here, we set out to determine the discriminatory ability of two of the more commonly used miRNA detection methods: the Exiqon LNA miRCuRY quantitative polymerase chain reaction (qPCR) and the ThermoFisher Taqman microRNA qPCR. Neither Exiqon nor ThermoFisher claim that these commercial qPCR methods are capable of discriminating individual isomiRs. However, we set out to extend on demonstrated and advertised success in discriminating closely related variants of hsa-let-7 [18]. We wanted to determine whether such discriminatory capacities extend to isomiRs with single nucleotide endpoint differences, by extending previous attempts to address this question [19]. Using four isomiRs from the miR-21-5p locus, we investigated the capacity of these assays to detect these miRNA isoforms, using synthetic RNAs and total RNA extracted from model cell lines.

## 2. Results

### 2.1. Hsa-miR-21-5p isomiRs

**Figure 1:**
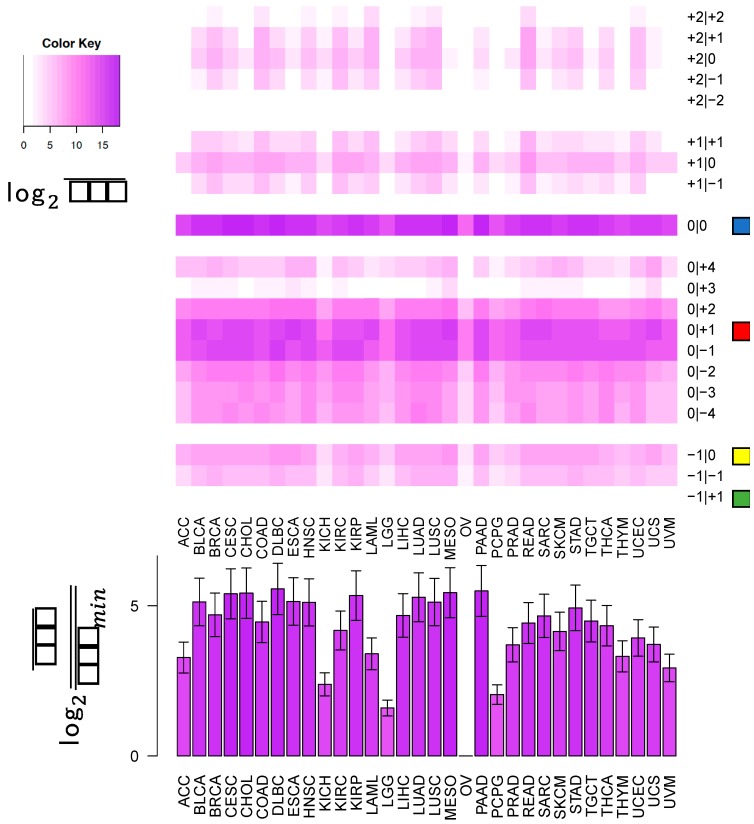
Heatmap of short RNA-seq mean RPM values across 32 TCGA projects. To assess the level of miR-21-5p isomiRs that are closely related to hsa-miR-21-5p 0|0, we calculated the mean abundance of *n* = 20 isomiRs across *n* = 32 TCGA projects. The mean was calculated using all sample types available in each project (specifically: tumor, normal, blood based, or metastasis). We plot a heatmap, where the intensity of purple in each cell represents the log2 mean RPM for that isomiR in that cancer. isomiRs are labeled by the color with which we denote them in later experiments (right). The lower panel shows the log2 fold difference between the level of hsa-miR-21-5p 0|0 in each TCGA project versus the absolute minimum mean observed in ovarian cancer (OV) (mean RPM = 6382.92). Errors bars show propagated formula for the Standard Error of the Mean.

Of all characterized miRNAs, miR-21-5p is one of the most well studied. There are currently 233 reported interactions between miR-21-5p and 103 unique genes in miRTarbase [20]. With the use of the vast resource of The Cancer Genome Atlas (TCGA) (http://cancergenome.nih.gov), we were able to take a closer look at the expression of the miR-21-5p locus across 10,274 samples representing 32 distinct tissue types. In total, there are 90 distinct isomiRs of hsa-miR-21-5p expressed in the TCGA dataset. Figure 1 shows a heatmap of the mean log2 Reads Per Million (RPM) normalized read count of each of 20 isomiRs of hsa-miR-21-5p that can be recovered from the TCGA. These isomiRs were subselected due to their high sequence similarity to the 0|0 isomiR. From this heatmap, it is clear that the canonical isomiR (0|0 isomiR) is expressed at a higher level than its closely related counterparts. Interestingly, as can be observed, there is a range in expression values for the different isomiRs, but some isomiRs are present in only a subset of the tissue types, indicating tissue specific functions or biogenesis. This is further highlighted in the lower panel of the figure, which shows that the miR-21-5p 0|0 isomiR is expressed over a large range of values in each of the 32 tissues we surveyed.

### 2.2. Case 1: Assaying one isomiR at a Time/H_2_O Milieu

Above, we show that miR-21-5p produces many isomiRs with varying expression profiles and differences across tissue types. As these observations were made using RNA-sequencing, it raises the question of whether we would be able to quantify the expression profiles of specific isomiRs using standard qPCR methods.

In testing the discriminatory capacity of qPCR methods, we rely on two assumptions: (1) that the behavior of these methods is independent of the miRNA sequence being quantified; and (2) that, by extension, the behavior of these methods is independent of the isomiR sequence being quantified. With these assumptions in mind, we specifically chose four very closely related isomiRs of miR-21-5p (Table 1) with which to examine the discriminatory capacity of two qPCR methods. In addition to the canonical (0|0) isomiR, three other isomiRs were selected according to the following logic: 0|+1: this is the next most abundant isomiR of hsa-miR-21-5p; −1|0: this is a 5′ isomiR that is closely related to 0|0 in sequence, but is present at a level several orders of magnitude lower than 0|0 in human tissue, though it is present in every human tissue assayed; −1|+1: this isomiR is absent from the short RNA-seq data for human tissue, though it is closely related in sequence to the other three isomiRs with which we worked.

**Figure 2:**
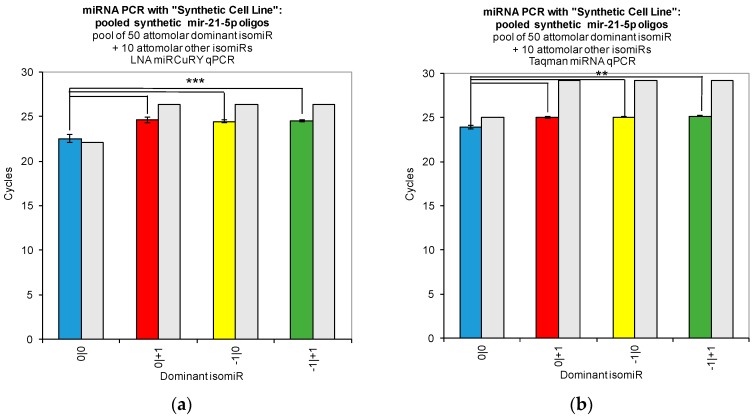
miRNA qPCR with pooled synthetic oligo RNAs: “synthetic cell lines.” To determine the specificity of both Exiqon LNA miRCuRY PCR and ThermoFisher Taqman miRNA qPCR, we combined the four isomiRs of hsa-miR-21-5p (Table 1) such that we formed four pools in which each one of the isomiRs in turn was present at five times the concentration of the other three. We then performed one RT and three qPCR replicates with each technique. Panel (a) shows the results for LNA qPCR, while panel (b) shows the results for Taqman qPCR. Colored bars show the Ct value for each of the synthetic pools, while grey bars show the expected Ct value for each pool. Three stars (***) indicate a *p*-value ≤ 0.001, while two stars (**) indicate a *p*-value ≤ 0.01.

We worked with two commercial qPCR assays: Exiqon LNA miRNA PCR (Exiqon, Woburn, MA, USA) and ThermoFisher Taqman miRNA PCR (ThermoFisher, Waltham, MA, USA). Using synthetic oligonucleotides for the four isomiRs (Table 1), we first calibrated our qPCR technique by building standard curves with serial dilutions of synthetic RNA. It should be noted that these are “off the shelf” qPCR probes purchased from Exiqon and ThermoFisher, designed for the miRBase entry for hsa-miR-21-5p, which we know to be the 0|0 isomiR. In these standard curves, we were able to discriminate the dilution of the hsa-miR-21-5p 0|0 isomiR, observing a relatively consistent increase in Ct value for each step in the serial dilution (Figure 2). For each of the other three hsa-miR-21-5p isomiRs that we studied, we were not able to observe a difference in signal over the range of serial dilution. Nonetheless, the observation that Ct values were observed (Ct value of ~30 for LNA ((Figure 2a) and Ct value of ~25 for Taqman (Figure 2b)) does suggest that some amplification of the RNA is occurring. We also ran controls using pure H_2_O as both a template for the initial reverse transcriptase (RT) reaction and as a template for the qPCR reaction, each of which showed “Undetected” Ct values on analysis (data not shown). These data indicate that signals observed in the standard curve experiment are derived from interaction of the qPCR probes with the synthetic RNA oligonucleotides and that some crosstalk between different isomiRs does occur.

### 2.3. Case 2: Assaying Multiple isomiRs Simultaneously/H_2_O Milieu

To further simulate the “real-world” environment, we combined the four isomiRs into four pools, representing synthetic cell lines. We chose our concentrations to mirror the ground truth observed in the TCGA (bottom panel of Figure 1). Three cancers: Kidney Chromophobe Carcinoma (KICH) (mean RPM = 33,225.42), Lower Grade Glioma (LGG) (mean RPM = 19,349.38) and Paraganglioma and Pheochromocytoma (PCPG) (mean RPM = 26,432.04), have mean RPM that is within 5 times greater than the minimum observed value for hsa-miR-21-5p 0|0 in the TCGA (ovarian cancer (OV), mean RPM = 6382.92). These are the next highest levels of miR-21-5p 0|0 that we observed. We therefore assembled four synthetic pools, each of which was marked by having 5× the concentration of a single isomiR over the concentration of that isomiR in the other three pools. This design allowed us to determine whether we would be able to detect such a small quantitative difference in the presence of significant background, while controlling for the total amount of RNA in each pool. In other words, can the qPCR probes discriminate isomiRs that are present at differential abundances with respect to one another? We hypothesized that the synthetic pool in which hsa-miR-21-5p 0|0 was present at five times the concentration of the other isomiRs would show a Ct value that was statistically significantly different from the Ct values for an H_2_O only sample or from the other three synthetic pools.

**Figure 3:**
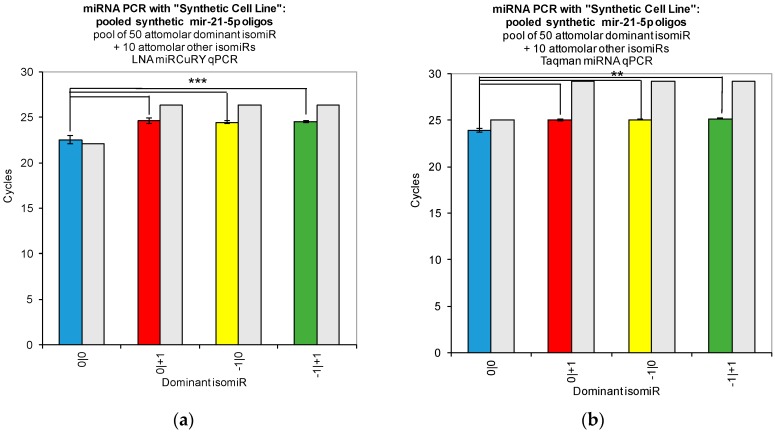
miRNA qPCR with pooled synthetic oligo RNAs: “synthetic cell lines.” To determine the specificity of both Exiqon LNA miRCuRY PCR and ThermoFisher Taqman miRNA qPCR, we combined the four isomiRs of hsa-miR-21-5p (Table 1) such that we formed four pools in which each one of the isomiRs in turn was present at five times the concentration of the other three. We then performed one RT and three qPCR replicates with each technique. Panel (a) shows the results for LNA qPCR, while panel (b) shows the results for Taqman qPCR. Colored bars show the Ct value for each of the synthetic pools, while grey bars show the expected Ct value for each pool. Three stars (***) indicate a *p*-value ≤ 0.001, while two stars (**) indicate a *p*-value ≤ 0.01.

In Figure 3, we plot the Ct value observed for each of the four pools (colored bars) together with the expected Ct value if only the hsa-miR-21-5p 0|0 synthetic RNA in each pool was amplified (grey bars). Firstly, the pool in which 0|0 was dominant does differ significantly from the other three pools in the case of both LNA and Taqman miRNA qPCR (Figure 3a,b). Specifically, p valuep-values for an unpaired t test comparing LNA qPCR results the 0|0 (blue) pool are as follows: vs. 0|+1 (red) *p* = 0.0031, vs. −1|0 (yellow) *p* = 0.0025, vs. −1|+1 (green) *p* = 0.0019. *p*-values for an unpaired *t* test comparing Taqman qPCR results the 0|0 (blue) pool are as follows: vs. 0|+1 (red) *p* = 0.0007, vs. −1|0 (yellow) *p* = 0.0003, vs. −1|+1 (green) *p* = 0.0002. These results indicate that both methods remain sensitive to detection of concentration differences in the presence of background.

Secondly, our observed Ct values differ from those that we expect for amplification of hsa-miR-21-5p 0|0 alone. We computed the expected Ct value in each case by fitting a linear equation to the 0|0 standard curve in Figure 2 and then computing the Ct value for both 12.5 attomolar and 60 attomolar 0|0, as was used in the synthetic pools. Specifically, we compute the following expected Ct values: miRCuRY 0|0: 22.07, miRCuRY 0|+1: 26.32, miRCuRY −1|0: 26.32, miRCuRY −1|+1: 26.32; Taqman 0|0: 25.06, Taqman 0|+1: 29.17, Taqman −1|0: 29.17, Taqman −1|+1: 29.17. For LNA qPCR, the observed Ct for the pool in which 0|0 is dominant is higher than the expected Ct value for 0|0 alone (Figure 3a), indicating that the background RNA detracted from the efficacy of amplification of 0|0. For ThermoFisher qPCR, all of the synthetic pools show a Ct value lower than that expected (Figure 3b), indicating that background RNAs are contributing to the observed Ct. These results indicate that although these assays remain sensitive in the presence of background, there is substantial cross-reactivity in detection of individual isomiRs.

### 2.4. Case 3: Assaying Single isomiRs Following Transfection into a Model Cell Line

We next sought to determine whether the observed cross-reactivity is limited to synthetic contexts only. To this end, we repeated the qPCRs following transfection of each isomiR mimic in turn into HEK293T cells. We transfected one of four miRVana miRNA mimics ordered from ThermoFisher, to artificially alter the abundance of hsa-miR-21-5p isomiRs: 0|0, 0|+1, −1|0, −1|+1. Transfections were performed in triplicate for each of the five transfected RNAs (including a short scrambled negative control sequence). We were unable to recover RNA from one replicate of both 0|0 and 0|+1 transfections.

**Figure 4:**
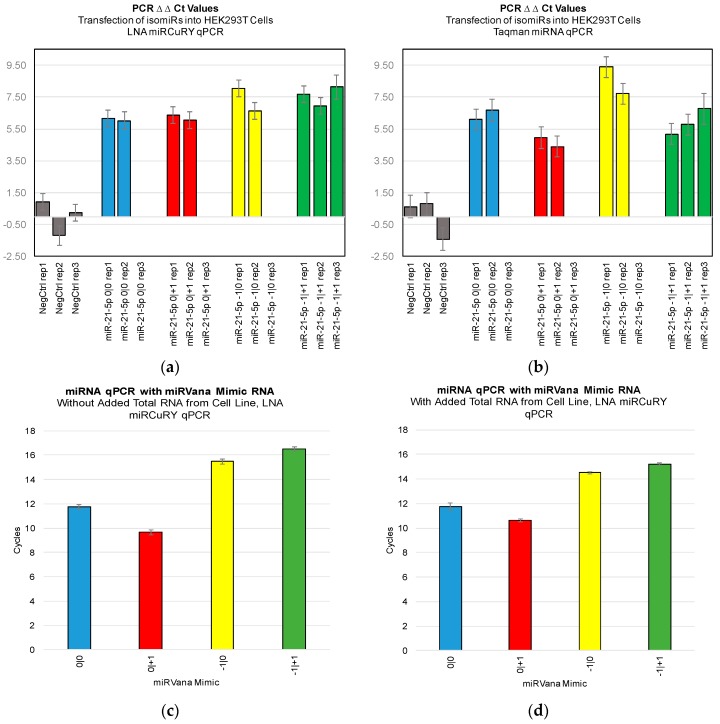
miRNA qPCR detection with transfected HEK293T cells. To determine the specificity of both Exiqon LNA miRCuRY PCR (**a**) and ThermoFisher Taqman miRNA qPCR (**b**), we transfected miRNA mimics of each of the four isomiRs of hsa-miR-21-5p that we studied into HEK293T cells in triplicate (see Table 1). We then performed one RT and three qPCR replicates with extracted RNA from each of the transfections. Panel (**a**) shows the 2^−∆∆Ct^ values for the Exiqon LNA method, calculated using the average of the three negative control experiments as the reference for the delta-delta Ct calculation. Panel (**b**) shows the same calculation for the ThermoFisher miRNA qPCR experiment. Panels (**c**) and (**d**) serve as quality control for the transfection. Panel (**c**) shows the results of Exiqon LNA method qPCR after using each miRVana mimic alone as template RNA for the RT reaction. Panel (**d**) shows the results of Exiqon LNA method qPCR after using each miRVana mimic, together with total RNA extracted from MD-MB-468 cells, as template RNA for the reverse transcriptase (RT) reaction.

In this experiment, we were unable to distinguish the cell line in which hsa-miR-21-5p 0|0 abundance was altered to become differentially abundant with either method. Instead, all four transfected cell lines and their replicates showed a decreased Ct value with respect to a negative control, with both LNA and Taqman qPCR. Differences in RNA extraction efficiency do not account for this result - see results normalized to a U6 snRNA control and scrambled short RNA transfection, using the delta-delta-Ct (∆∆Ct) method (Figure 4a,b). To compute the ∆∆Ct, we first subtract the value of U6 snRNA from our hsa-miR-21-5p-values and subsequently subtract the delta Ct of the average of all three negative control experiments. We then plot 2^−∆∆Ct^. Figure 4a,b shows that all four transfected RNAs appear to be detected by the hsa-miR-21-5p 0|0 qPCR probes. Contrary to our hypothesis, we do not observe a statistically significant increased detection of transfected hsa-miR-21-5p 0|0; rather, we observe a substantial increase in signal when transfecting all four isomiRs. Taken together, the results from both synthetic and real cell lines indicate that the two qPCR methods are unable to sufficiently discriminate between differentially abundant isomiRs.

### 2.5. Differential Abundance of 5′ isomiRs

**Figure 5:**
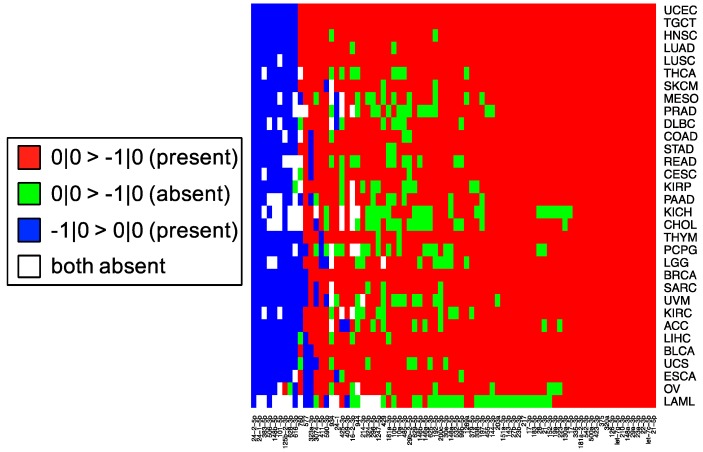
Comparison of the sign of the ratio of the 0|0 to the −1|0 across The Cancer Genome Atlas (TCGA). We analyzed 10,274 short RNA-seq datasets and found that 223 mature miRNAs show expression for both 0|0 and −1|0 isomiRs in at least 1 of the 32 TCGA projects. We then compared the mean RPM of these isomiRs and pooled our observations of 223 isomiR pairs across 32 cancers into four categories: white: both isomiRs are absent from this project; blue: the −1|0 isomiR is more abundant than the 0|0; red: the 0|0 isomiR is more abundant than the −1|0, which is also present; green: the 0|0 isomiR is more abundant than the −1|0, which is entirely absent. We plot the resultant observations as a heatmap, showing that most mature miRNAs predominantly express the 0|0 isomiR at high abundance across the TCGA, but that a significant portion show the reverse pattern of expression.

As we showed in Figure 1, the 0|0 isomiR of hsa-miR-21-5p is the most abundant among those closely related in sequence. We sought to determine if this was the case for other miRNAs present in the TCGA. We compared the expression profiles for two 5′ isomiRs (the 0|0 and −1|0) for 223 miRNA loci that expressed both of these isomiRs in TCGA. Of these 223, 78 miRNAs show expression of both the 0|0 and the −1|0 in more than two thirds of the TCGA projects tested. We plot the sign of the ratio of the mean Reads Per Million (RPM) across samples in each of 32 projects for these 78 miRNAs (Figure 5). The majority of these miRNAs show higher expression of the 0|0 isoform than the −1|0 isoform (red in Figure 5); a large portion even express the 0|0 alone in the absence of −1|0 in some cancers (green in Figure 5). Most interestingly, a subset of the miRNAs do have the −1|0 isoform as the most abundantly expressed (blue in Figure 5). These findings raise the possibility that studies of canonical miRNAs in specific tissues may not capture the most relevant and functional sequence.

## 3. Discussion

In this study, we sought to determine whether two commonly-used qPCR assays can discriminate amongst isoforms of the same miRNA. The two approaches tested here: LNA and Taqman qPCR: are widely used. Being able to efficiently and accurately quantify isomiR abundance is fast becoming important as researchers begin to delve into studies of the isomiRs’ functional and diagnostic capacities. To assess this discriminatory capacity, we characterized the ability of these methods to quantify differences in both 3′ and 5′ hsa-miR-21-5p isomiRs, when these isomiRs are in isolation and when these isomiRs are in combination in both synthetic and real cell contexts. miR-21 was chosen for this study, because it is ubiquitous and shows high expression across a wide range of tissue types (Figure 1). miR-21-5p is also one of the most widely characterized miRNAs with reported functions in diverse contexts [19]. The specific isomiRs of miR-21-5p were chosen for their high sequence similarity, because they represent the range of abundances (Figure 1) over which miR-21-5p isomiRs are found, from most abundant to completely absent.

Our work reveals large variations in the assays’ ability to detect changes in the concentration of individual isomiRs (Figure 2). Both tested qPCR assays showed greatest sensitivity for detection of the isomiR sequence for which they were originally designed (0|0 isomiR) (Figure 2). In Figure 2, we show that the two qPCR assays are able to detect concentration differences in the sequences for which they are designed, as evidenced by the fact that we were able to recover a standard curve for the hsa-miR-21-5p 0|0 synthetic RNA dilutions with each qPCR method. However, use of these probes to assay synthetic RNA representing any of the closely related isomiRs reveals that these structural variants do contribute to qPCR signals coming from these probes, as evidenced by the non-zero Ct signal observed for the alternate standard curves (Figure 2). Moreover, when we combined multiple isomiRs into a “synthetic” cell line, we observed significant cross-reactivity between closely related isomiRs and the qPCR probes designed only for the canonical isomiR (0|0) (Figure 3). Furthermore, we were not able to discriminate cell line RNA in which we transfected 0|0 isomiR from those in which we transfected these same closely related isomiRs (Figure 4a,b). Importantly, these differences can be attributed to detection of the transfected RNA, as qPCR of the transfected RNA alone: in the presence and absence of total RNA from cell lines: showed a high Ct signal for each of the four isomiRs (Figure 4c,d).

Our findings suggest that traditional qPCR assays are not adequately specific to quantify distinct miRNA isomiRs. This should not be surprising. Both LNA and Taqman miRNA qPCR assays have been shown to be highly specific for miRNA isomiRs that differ on internal base pairs, such as the let-7 family. For example, recent work showed that these qPCR methods possess exceptional discriminatory capacity for internal sequence differences, giving rise to their ability to distinguish individual miRNAs in the whole cell milieu [18]. We conjecture that this specificity arises from the fact that internal base differences are disruptive to probe-target interactions. However, when the differences lie solely at the ends of the target sequences, the full discriminatory power of these assays is diminished due to flexibility in the structures of target-probe pairs. Consequently, changes in the abundance of isomiR variants whose sequences differ only slightly from those of the archetype miRNA cannot be quantified using the current incarnation of these assays. Moreover, since the assays appear to be optimized for the 0|0 isomiR, any changes in the abundance of nearly-similar isomiRs will manifest themselves as “cross-talk” that will skew the quantification of the 0|0 isomiR. We believe these results hold for the qPCR probes designed for the 0|0 isomiR, and infer that qPCR probes designed for other isomiRs will exhibit the same tendency to cross-react, rendering testing of alternate probes superfluous.

This cross-reactivity is highlighted by the fact that the −1|+1 isomiR is not an endogenously expressed isomiR in any of the 32 TCGA tissues profiled (Figure 1). Nonetheless, the qPCR probes tested are capable of detecting artificially elevated levels of this rare isomiR. In fact, a substantial background signal is observed in the 0|0 probe signal, both in synthetic and real cell contexts, before and after transfection of this isomiR (Figure 2, Figure 3 and Figure 4). If one were to attempt to probe −1|+1 levels using a qPCR probe, in order to confirm its absence from a sample and thereby heighten suspicion of a specific disease (see [16] for example), one would potentially encounter background signals that render the problem of confirming lack of a specific isomiR non-trivial. The reverse is true as well; in order to confirm the presence of a specific isomiR, one would need to be sure that any signal observed on qPCR captured the abundance of the isomiR itself, and not closely related sequences, which here are shown to produce non-trivial background noise.

These observations raise an important question for researchers: when preforming miRNA qPCR assays, what molecule is being profiled and what is that molecule’s true abundance? Our experiments indicate that individual LNA and Taqman probes will be influenced by “cross-talk” and in actuality, profile multiple isomiRs among those that are expressed from a distinct miRNA arm, rather than a single isomiR. As we have shown previously [11] and in Figure 5, the most abundant isomiR is not always the 0|0 isomiR, and in many cases, these differences will vary based upon the tissue type. Moreover, recent findings suggest that specific isomiRs will change in a manner that is correlated with an individual’s sex and race as well as the nature of the tissue at hand, the tissue’s state, and the disease subtype [11,16]. However, in light of our findings, we reason that these investigations would be exceptionally difficult to verify using these commercial qPCR methods.

IsomiRs have also recently been described in functional roles that are drastically different from those of the mature miRNA to which they are most closely related. We showed that isomiRs of miR-183-3p only share 15% of their differentially affected targets after transfection [10]. Furthermore, other groups have elucidated functional roles for isomiRs of miR-140-3p [13], miR-375 [17] and miR-142-3p [12] that differ from those roles established for the mature miRNA. Taken together, these observations have set the precedent that different isomiRs from the same locus will have functionally distinct roles. While much more work is needed to functionally evaluate the role of different isomiRs, being able to experimentally distinguish amongst them is of great importance. Our observations suggest that the two commercial qPCR technologies tested here have limited ability to distinguish amongst various isomiRs.

While our work reveals some of the limitations of commercial miRNA qPCR assays, one alternative approach to address this problem has been explored. This method, named “dumbbell PCR” (dbPCR) [21], relies on the addition of a second stem-loop PCR step to the 5′-end of the isomiR, in order to improve the discriminatory capacity of the traditional Taqman qPCR method. While the authors do demonstrate the ability to detect both 5′ and 3′ isomiRs of miR-16, they do not compare their method to commercial qPCR assays. Such a method represents a promising tool for use in future experiments with isomiRs. However, despite the potential utility of dbPCR, it comes with the limitation of the added expense of custom-made qPCR probes and time to optimize the PCR reactions.

As the idea of functional roles of isomiRs gains traction, being able to accurately, specifically and inexpensively profile these molecules will become even more pressing. We recognize that sequencing approaches provide the technical specificity necessary to confidently capture sequences that differ in their 5′ or 3′ endpoints, but simultaneously understand that such approaches are not always feasible because of the high costs and relative difficulty in the analysis of the generated data. However, while we look forward to the next generation of short RNA qPCR methods, we believe that the safest current approach to the study of isomiRs is short RNA sequencing.

## 4. Materials and Methods

### 4.1. Synthetic RNAs

Four synthetic RNA oligonucleotides (Integrated DNA Technologies, Coralville, IA, USA), representing the four hsa-miR-21-5p isomiRs were designed (Table 1). Each synthetic RNA oligonucleotide bore standard modifications as are seen in the cell: a 5′-phosphate group with a 3′-hydroxyl group. We created four “synthetic cell lines” from these isomiRs, by combining 50 microliters of a 10^–14^ M starting concentration of the “dominant” isomiR together with 10 microliters of a 10^–14^ M starting concentration of the other three isomiRs, in H_2_O. We repeated this process an additional three times to create a synthetic cell line with each of the four isomiRs being “dominant” in turn.

### 4.2. qPCR Assays

We performed qPCR according to manufacturer specifications, using two popular qPCR methods: LNA miRCuRY (Exiqon, Woburn, MA, USA) and Taqman (ThermoFisher, Waltham, MA, USA). All assays were run on an Applied Biosystems StepOne qPCR machine. For both LNA and Taqman, we performed qPCR using the commercially available probe from each company that is marketed as detecting hsa-miR-21-5p. These probes are designed to detect the canonical hsa-miR-21-5p sequence, or the 0|0 isomiR.

For each qPCR assay, we performed a standard curve experiment of serial dilutions of each isomiR, from a starting concentration of 10–13 M (100 fm) RNA stepping by a dilution factor of 10 down to a concentration of 10–18 M (1 am). This range was selected to represent the physiological range of isomiR concentrations we have previously observed in sequencing data (not shown). For each synthetic oligo dilution, qPCR was run in triplicate from a single reverse transcriptase (RT) reaction.

LNA miRCuRY qPCR and Taqman miRNA qPCR were then repeated as above, using each of these synthetic cell lines as starting material and using the qPCR probe designed for the 0|0 isomiR in each case. Triplicate qPCR was run from a single RT starting sample.

### 4.3. Cell Culture and Transfection

To assess the discriminatory capacity of these assays in real cell contexts, we transfected mimics of each of the four isomiRs into HEK293T cells. Two of the mimics (hsa-miR-21-5p 0|0 and 0|+1) were purchased directly from ThermoFisher (ThermoFisher, Waltham, MA, USA), while the other two isomiRs (hsa-miR-21-5p −1|0 and −1|+1) were custom ordered. For comparison, we also transfected a short RNA negative control of a scrambled RNA sequence. Transfections were performed using Lipofectamine RNAi-Max. The cells were exposed to transfection reagents for 5 h prior to removing the transfection medium and replacing it with fresh Dulbecco Modified Eagle Medium (DMEM) supplemented with 10% FBS and penicillin/streptomycin. For each transfection, we used 1 microliter of 50 micromolar stock. After 48 h, we extracted RNA and performed LNA miRCuRY and Taqman miRNA assays as above. Transfection was performed in triplicate: each transfection yielded a single RT sample with which qPCR was run in triplicate. For normalization, we ran Taqman miRNA qPCR using a U6 probe (ThermoFisher, Waltham, MA, USA). We performed cell culture using standard techniques.

### 4.4. Short RNA-Seq Data Analysis

In order to discuss the implications of our qPCR findings, we downloaded and analyzed 10,274 short RNA-seq samples from TCGA, corresponding to samples that have not been marked for removal from one of the 32 TCGA projects due to incorrect filing (these samples are annotated as such in the file_annotations.txt file included with each download). We downloaded these data through the online data portal at: http://gdc-portal.nci.nih.gov. We then computed isomiR expression using the distributed miRNA isoform data, as in [11,16], and further subjected the computed reads to Threshold-seq, in order to determine a dynamic threshold for inclusion of isomiRs in downstream analysis [22]. Finally, we built expression matrices for isomiRs from each of the 32 TCGA projects, retaining only those isomiRs within a specific TCGA project that exceed Threshold-seq filtering level in at least one sample and retaining samples of any original type (specifically: tumor, normal, blood based, or metastasis). We subsequently computed the mean expression of isomiRs using these matrices, and plotted the results using the heatmap.2() function of the gplots package in R.

## Figures and Tables

**Table 1:** isomiR variants of hsa-miR-21-5p. Briefly, the nomenclature shorthand representation for the isomiRs is represented relative to the archetype sequence (sequence annotated in public databases such as miRBase, labeled as “0|0”): e.g., the “−1|+1” indicates the isomiR whose 5′ terminus begins one position to the left (−1) of the archetype’s 5′ terminus and whose 3′ terminus ends one position to the right (+1) of the archetype’s 3′ terminus. In order to explore the specificity of miRNA qPCR methods for isomiR endpoint detection, we assayed synthetic RNA oligonucleotides of four hsa-miR-21-5p isomiR variants. miRVana mimics matching these isomiR sequences were ordered from ThermoFisher. We ordered synthetic RNA oligonucleotides with 5′-phosphate and 3′-hydroxyl groups from Integrated DNA Technologies.

isomiR	Sequence
hsa-miR-21-5p 0|0 (miRBase Entry)	UAGCUUAUCAGACUGAUGUUGA
hsa-miR-21-5p 0|+1	UAGCUUAUCAGACUGAUGUUGA**C**
hsa-miR-21-5p −1|0	**G**UAGCUUAUCAGACUGAUGUUGA
hsa-miR-21-5p −1|+1	**G**UAGCUUAUCAGACUGAUGUUGA**C**
